# Influence of severity of infection on the effect of appropriate antimicrobial therapy for *Acinetobacter baumannii* bacteremic pneumonia

**DOI:** 10.1186/s13756-020-00824-4

**Published:** 2020-09-29

**Authors:** Fang-Yu Kang, Chorng-Kuang How, Yung-Chih Wang, Aristine Cheng, Ya-Sung Yang, Shu-Chen Kuo, Chang-Pan Liu, Yuag-Meng Liu, Te-Li Chen, Yi-Tzu Lee

**Affiliations:** 1 Department of Emergency Medicine, Taipei Veterans General Hospital, No.201, Section 2, Shipai Road, Taipei, 11217 Taiwan; 2 Faculty of Medicine, School of Medicine, National Yang-Ming University, Taipei, Taiwan; 3 Division of Infectious Diseases and Tropical Medicine, Department of Internal Medicine, Tri-Service General Hospital, National Defense Medical Center, Taipei, Taiwan; 4 Department of Internal Medicine, National Taiwan University Hospital and National Taiwan University College of Medicine, Taipei, Taiwan; 5 National Institute of Infectious Diseases and Vaccinology, National Health Research Institute, Miaoli County, Taiwan; 6 Division of Infectious Diseases, Department of Internal Medicine, Mackay Memorial Hospital, Taipei, Taiwan; 7 Department of Medical Research, Mackay Memorial Hospital, Taipei, Taiwan; 8 Division of Infectious Diseases, Department of Internal Medicine, Changhua Christian Hospital, Changhua, Taiwan; 9 Graduate Institute of Life Sciences, National Defense Medical Center, Taipei, Taiwan; 10 Division of Infectious Diseases, Department of Medicine, Taipei Veterans General Hospital, Taipei, Taiwan

**Keywords:** Appropriate antimicrobial therapy, *Acinetobacter baumannii*, Bacteremia, Pneumonia, Severity

## Abstract

**Background:**

The impact of appropriate antimicrobial therapy for *A. baumannii* bacteremic pneumonia has not been well established due to the inclusion of the three phenotypically indistinguishable *Acinetobacter* species and confounding factors including underlying diseases and severity of infection. This retrospective study aimed to evaluate the impact of appropriate antimicrobial therapy on 14-day mortality in *A. baumannii* bacteremic pneumonia patients after adjusting for risk factors.

**Methods:**

This study was conducted at five medical centers in Taiwan between July 2012 and June 2016. *A. baumannii* species identification was performed using reference molecular methods. Risk factors for 14-day mortality were analyzed via logistic regression. The interaction between the Acute Physiology and Chronic Health Evaluation (APACHE) II score and appropriate antimicrobial therapy was assessed using the logistic model.

**Results:**

A total of 336 patients with monomicrobial *A. baumannii* bacteremic pneumonia were included in this study. The overall 14-day mortality rate was 47.3%. The crude mortality of appropriate antimicrobial therapy was 35.9% (57 of 151 patients). Appropriate antimicrobial therapy was associated with a lower mortality after multivariate adjustment (odds ratio [OR], 0.57; 95% confidence interval [CI], 0.34–0.97; *p* = 0.04), and the effect was influenced by APACHE II score (OR for interaction term, 0.0098; 95% CI, 0.0005–0.1885; *p* = 0.002). Further analysis demonstrated that appropriate antimicrobial therapy significantly reduced 14-day mortality among the patients with an APACHE II score > 35 (OR 0.0098; 95% CI 0.0005–0.1885).

**Conclusion:**

Appropriate antimicrobial therapy decreases 14-day mortality of the most severely ill patients with *A. baumannii* bacteremic pneumonia.

## Background

Nosocomial pneumonia is one of the main causes of mortality and morbidity among hospitalized patients [1, 2]. The estimated attributable mortality ranges between 33 and 50% [3]. *Acinetobacter baumannii* is one of the leading pathogens of nosocomial pneumonia worldwide and is associated with poorer outcomes [4–7]. Although it is difficult to determine the attributable mortality due to severe comorbidities [5, 8], many studies have shown that the high levels of resistance of *A. baumannii* to antimicrobials may play an important role [8–12]. The high antimicrobial resistance of *A. baumannii* leads to higher rates of inappropriate empirical antimicrobial therapies, and may contribute to a greater risk of death [13, 14].

Numerous studies have investigated the efficacy of antimicrobial therapy for *A. baumannii* bacteremia [15–18]. Appropriate antimicrobial therapy is associated with a lower mortality rate in *A. baumannii* bacteremia patients [15, 17], and the therapeutic effects might be more significant in severely ill patients [18]. The impact of appropriate antimicrobial therapy for *A. baumannii* bacteremic pneumonia has not been well established. Many factors contribute to the mortality of *A. baumannii* bacteremic pneumonia, and it remains unclear whether appropriate antimicrobial therapy increases survival among all patients or only among patients with certain demographic or clinical characteristics. Furthermore, the association between appropriate antimicrobial therapy and mortality for *A. baumannii* bacteremic pneumonia has been difficult to establish due to the confounding influence of the three phenotypically indistinguishable *Acinetobacter* species that make up the *A. baumannii* (Ab) group (*A. baumannii*, *Acinetobacter nosocomialis* and *Acinetobacter pittii)*. The aims of this retrospective study were to evaluate the impact of appropriate antimicrobial therapy on the 14-day mortality in genomically identified *A. baumannii* bacteremic pneumonia patients, and to determine if the therapeutic effect of appropriate antimicrobial therapy differed between patients with different infection severities.

## Materials and methods

### Data collection and patients

This study was conducted at the following five medical centers in Taiwan: Taipei Veterans General Hospital (TVGH, 2900 beds), Tri-Service General Hospital (TSGH, 1712 beds), Mackay Memorial Hospital (MMH, 2055 beds), and National Taiwan University Hospital (NTUH, 2200 beds) in Northern Taiwan and Changhua Christian Hospital (CCH, 1676 beds) in Central Taiwan. Data was collected between July 2012 and June 2016. The inclusion criteria for *A. baumannii* bacteremic pneumonia were: (1) ≥1 positive blood culture for *A. baumannii* which could not be attributed to an infection source other than the lower respiratory tract; (2) a clinical course compatible with the diagnosis of pneumonia, including a new pulmonary infiltrate plus one additional criterion (fever ≥38 °C, blood leukocytosis ≥10,000 cells/mm^3^ or leucopenia ≤3000 cells/mm^3^), together with one or more of the following conditions: new cough, change in sputum color, chest pain, and dyspnea; (3) ≥1 positive respiratory sample (sputum, endotracheal aspirate, or broncho-alveolar lavage [BAL]) for *A. baumannii* collected within 48 h before or after the first positive blood culture for *A. baumannii*. Patients below 20 years of age or without a complete medical record were excluded. The study protocol was approved by the Research Ethics Committee of all participating hospitals. Informed consent was waived because of the retrospective nature of the study and the analysis used anonymous clinical data.

### Study variables and definitions

The following data were collected from patient’s medical records: demographic information, comorbid conditions, duration of hospital and intensive care unit (ICU) stays, time, dose and route of antimicrobial therapy, the use of ventilator, and procedures (central venous catheters, arterial catheters, foley catheter, nasogastric tube, hemodialysis, and tracheostomy) at the time of bacteremia.

The onset of bacteremia was defined as the day the positive blood culture of *A.baumannii* was collected. The bacteremic pneumonia was considered acquired in the ICU if the positive respiratory sample for *A.baumannii* and positive blood culture for *A.baumannii* were both obtained at least 48 h after ICU admission. Previous ICU admission was defined as being admitted to ICU within 4 weeks prior to the onset of bacteremia.

Immunosuppressive therapy was defined as receiving cytotoxic agents within 6 weeks, corticosteroids at a dosage equivalent to or higher than 15 mg of prednisolone daily for 1 week within 4 weeks, or other immunosuppressive agents within 2 weeks of the onset of bacteremia. Chronic kidney disease was defined as an estimate glomerular filtration rate < 60 mL/min/1.73 m^2^. Neutropenia referred to an absolute neutrophil count < 0.5 × 10^9^ neutrophils/L. Recent surgery was defined as undergoing an operation within 4 weeks of the onset of bacteremia. Previous ventilator use was defined as mechanical ventilation use for more than 3 days in the past 4 weeks. The severity of patient illness was evaluated using the Acute Physiology and Chronic Health Evaluation (APACHE) II score within 24 h before bacteremia onset.

Appropriate antimicrobial therapy was defined as administration of the antimicrobial agent to the pathogen susceptible in vitro, within 48 h after the onset of bacteremia, with an approved route and dosage appropriate for end organ(s) function. Antimicrobial therapy that did not meet this definition was considered inappropriate. Monotherapy with an aminoglycoside was also considered to be an inappropriate therapy. An antimicrobial agent (or antimicrobial agents)-based therapy was defined as treatment with the antimicrobial agent(s) alone or in combination with other antimicrobial agent(s). The colistin loading dose was 5 mg/kg colistin base activity, followed by 5 mg/kg/d colistin base activity divided over 8 or 12 h in patients with normal renal function. For those with impaired renal function, the dosage was adjusted according to renal function as previously described [19, 20]. The loading dose of tigecycline was 100 mg, followed by a maintenance dose of 50 mg every 12 h. The primary outcome was all-cause 14-day mortality after the onset of *A. baumannii* bacteremia.

### Microbiological studies

The presumptive identification of the isolates to the level of the *A. baumannii* complex was determined using the Vitek 2 system (bioMérieux). All *A. baumannii* complex bloodstream isolates were regrown from storage, identified to species level, and tested for their susceptibility to various antimicrobials. A multiplex polymerase chain reaction method was used to identify *A. baumannii* to the genomic species level [21]. Polymicrobial bacteremia was defined as the concurrent isolation of one or more microorganisms other than *A. baumannii* from blood. Antimicrobial susceptibility to ampicillin-sulbactam, ceftazidime, cefepime, piperacillin-tazobactam, imipenem, meropenem, ciprofloxacin, levofloxacin, and amikacin was determined by the agar dilution method according to Clinical Laboratory Standards Institute criteria. Colistin minimal inhibitory concentrations (MICs) were determined by the broth macrodilution method to problems arising from the fact that the surface charge on the polystyrene microplate applied during manufacturing influences the level of colistin adsorption to the plate surface [22, 23] Tigecycline MICs were determined by the broth microdilution method using fresh medium [24]. Multidrug resistance (MDR) was defined as resistance to at least one agent in at least three of the following classes of antimicrobials: antipseudomonal cephalosporins, antipseudomonal carbapenems, ampicillin-sulbactam, fluoroquinolones, and aminoglycosides. Carbapenem resistance was defined as resistance to imipenem or meropenem. Extensive drug resistance (XDR) referred to non-susceptibility to imipenem or meropenem and all drug classes with the exception of colistin and tigecycline.

### Statistical analysis

Chi-squared test or Fisher’s exact test was used to compare categorical variables. The Student’s t test or Man-Whitney rank sum test was used to analyze continuous variables. Logistic regression models were used to assess independent risk factors for 14-day mortality. Biologically plausible variables which were significantly associated with mortality (*p* ≤ 0.05) in the univariable analysis were included in the multivariable analysis. Stepwise logistic regression was used. Interactions between the APACHE II score and the covariates were assessed in the logistic regression model. APACHE II scores were categorized into four groups (APACHE II score ≤ 15, > 15 and ≤ 25, > 25 and ≤ 35, > 35) in the logistic regression models based on their quartile distribution and previous studies [18, 25]. The time between bacteremia onset to mortality was analyzed using Kaplan-Meier survival analysis. A *p*-value < 0.05 was considered to be statistically significant. All the analyses were processed with Stata software version 12.

## Results

During the study period, 875 patients were found to have had at least one episode of bacteremia caused by *A. baumannii*. We excluded 164 patients with polymicrobial bacteremia and 375 patients with a positive blood culture attributable to another source of infection. A total of 336 patients who met the criteria of *A. baumannii* monomicrobial bacteremic pneumonia were included during the 4-year study period.

The overall 14-day mortality rate of *A. baumannii* bacteremic pneumonia was 47.3% (159 of 336 patients). The crude mortality of appropriate antimicrobial therapy was 35.9% (57 of 151 patients). The demographic and clinical characteristics are demonstrated in Table 1. The 14-day non-survivors were more likely to have hematological malignancies and have underwent immunosuppressive therapy but less likely to have cerebrovascular accident or recent surgery. Non-survivors had a significantly higher APACHE II score and higher rates of previous ventilator use. There was no significant difference in the rates of invasive procedures between the 14-day survivor and non-survivors.

**Table 1:** Demographic and clinical characteristics of patients with *Acinetobacter baumannii* bacteremic pneumonia stratified by 14-day mortality

Characteristics	Survivors (*n* = 177)	Non-survivors (*n* = 159)	*P*-value
Demographics			
Male, No. (%)	128 (72.3)	122 (76.7)	.36
Age, median (IQR), years	70 (67–72)	69 (66–72)	.64
Acquired in ICU, No. (%)	91 (51.4)	90 (56.6)	.34
Previous ICU admission	127 (71.8)	110 (69.2)	.61
Length of hospitalization before bacteremia, median (IQR), days	36 (24–48)	35 (27–44)	.98
Comorbidities, No. (%)			
Charlson co-morbidity score	3.8 (3.4–4.2)	3.9 (3.5–4.2)	.75
Malignancy	50 (28.3)	44 (27.7)	.91
Solid tumor	42 (23.7)	30 (18.9)	.28
Hematologic malignancy	8 (4.5)	21 (13.2)	.005
Type 2 diabetes mellitus	63 (35.6)	44 (27.7)	.12
Cerebrovascular accident	47 (26.6)	21 (13.2)	.002
Hypertension	85 (48.0)	65 (40.9)	.19
Immunosuppressant use	35 (19.8)	56 (35.2)	.001
Liver cirrhosis	19 (10.7)	14 (8.8)	.55
Chronic kidney disease	58 (32.8)	62 (39.0)	.23
Coronary artery disease	29 (16.4)	24 (15.1)	.75
Congestive heart failure	34 (19.2)	34 (21.4)	.62
Chronic obstructive pulmonary disease	39 (22.0)	33 (20.8)	.78
Collagen vascular disease	10 (5.7)	18 (11.3)	.06
Chemotherapy	13 (7.3)	21 (13.2)	.08
Neutropenia	8 (4.5)	13 (8.2)	.17
Recent surgery	64 (36.2)	32 (20.1)	.001
Invasive procedures, No. (%)^a^			
Arterial line	63 (35.6)	69 (43.4)	.14
Central venous catheter	111 (62.7)	113 (71.1)	.11
Hemodialysis	21 (11.9)	26 (16.4)	024
Tracheostomy	44 (24.9)	50 (31.5)	.18
Ventilator (previous use)	73 (41.2)	92 (57.9)	.002
Ventilator (current use)	121 (68.4)	121 (76.1)	.12
Ventilator associated pneumonia	121 (68.4)	113 (71.1)	.59
Clinical condition			
APACHE II score within 24 h before bacteremia, median (IQR)	23 (21–24)	33 (32–35)	<.001
Shock	75 (42.4)	75 (47.2)	.38
Resistance profiles of bloodstream isolates, No. (%)			
Multidrug resistance (MDR)^b^	156 (88.1)	150 (94.3)	.046
Carbapenem resistance	104 (58.8)	129 (81.1)	<.001
Extensive drug resistance (XDR)^c^	58 (32.8)	95 (59.8)	<.001
Appropriate antimicrobial therapy	94 (53.1)	57 (35.9)	.001

Abbreviations: *APACHE II* Acute Physiology and Chronic Health Evaluation II, *ICU* intensive care unit, *IQR* interquartile range

^a^At the time the blood culture was obtained

^b^Resistance to at least one agent in at least three of the following classes of antimicrobials: antipseudomonal cephalosporins, antipseudomonal carbapenems, ampicillin-sulbactam, fluoroquinolones, and aminoglycosides

^c^Extensive drug resistance (XDR) referred to non-susceptibility to imipenem or meropenem and all drug class except for colistin and tigecycline

The bloodstream isolates of non-survivors had higher MDR, XDR and carbapenem resistance rates than those of survivors (*p* < 0.05). Survivors were significantly more likely to have received appropriate antimicrobial therapy than non-survivors. Factors that significantly predicted 14-day mortality in logistic regression are shown in Table 2. Multivariable analysis showed that administration of appropriate antimicrobial therapy was independently associated with lower mortality (OR, 0.57; 95% CI, 0.34–0.97; *p* = 0.04). APACHE II score and XDR were independent predictors of 14-day mortality (both *p* < 0.001).

**Table 2:** Logistic regression of predictors for 14-day mortality in patients with *Acinetobacter baumannii* bacteremic pneumonia

	Univariable Analysis		Multivariable Analysis	
Characteristic	OR (95% CI)	*P*-value	OR (95% CI)	*P*-value
Hematologic malignancy	3.21 (1.38–7.48)	.004		
Cerebrovascular accident	0.42 (0.24–0.74)	.003	0.40 (0.20–0.81)	.011
Immunosuppressant use	2.21 (1.35–3.61)	.002	1.73 (0.95–3.16)	.072
Recent surgery	0.44 (0.27–0.73)	.001	0.50 (0.27–0.92)	.025
Carbapenem resistance	3.02 (1.84–4.96)	<.001		
Extensive drug resistance	3.05 (1.95–4.76)	<.001	3.19 (1.86–5.46)	<.001
Previous ventilator use	1.96 (1.27–3.02)	.002		
APACHE II score (categorical)	3.47 (2.57–4.68)	<.001	3.20 (2.33–4.39)	<.001
Appropriate antimicrobial therapy	0.49 (0.32–0.76)	.001	0.57 (0.34–0.97)	.04

All biologically plausible variables with a *p*-value < 0.05 in the univariable analysis were considered for inclusion in the logistic regression model in the multivariable analysis. A stepwise selection process was utilized. We found that only cerebrovascular accident, recent surgery, extensive drug resistance, APACHE II score, and appropriate therapy were statistically significant factors for 14-day mortality

Abbreviations: *APACHE II* Acute Physiology and Chronic Health Evaluation II, *CI* confidence interval

Further exploration of the potential effect modification on the impact of appropriate antimicrobial therapy on 14-day mortality suggested that the severity of infection is an effect modifier. Interactions between the APACHE II score and appropriate antimicrobial therapy were added to the logistic regression model. The interaction term was statistically significant (OR for interaction term, 0.0098; 95% CI, 0.0005–0.1885; *p* = 0.001). Table 3 demonstrates the adjusted ORs for appropriate antimicrobial therapy administered to four different severities of infection by APACHE II score categories. Appropriate antimicrobial therapy was not associated with a lower mortality among patients with APACHE scores ≤15 or > 15 and ≤ 25 or > 25 and ≤ 35 (Groups I, II, and III). On the other hand, among those with APACHE II scores > 35 (Group IV), appropriate antimicrobial therapy significantly reduced the 14-day mortality (OR 0.0098; 95% CI, 0.0005–0.1885). A similar magnitude of association and trend was also obtained when the APACHE II score was categorized into four groups based on its quartile distribution (Supplemental Table S1). Among patients with APACHE scores ≤35 (Groups I + II + III), appropriate antimicrobial therapy was not associated with a lower mortality by univariate and multivariate analysis (Supplemental Table S2). Subgroup analyses showed that among the patients who were admitted in the ICU at the time of bacteremia (101 patients), appropriate antimicrobial therapy lowers 14-day mortality in the patients with APACHE score > 35 (OR 0.023; 95% CI 0.0015–0.3508). Of the patients who were ventilator assisted at the time of bacteremia (242 patients), those with an APACHE score > 35 had a lower 14-day mortality rate if receiving appropriate antimicrobial therapy (OR 0.014; 05% CI 0.0007–0.2812).

**Table 3:** Adjusted odds ratios for appropriate antibiotics for 14-day mortality in patients with *Acinetobacter baumannii* bacteremic pneumonia: Stratified by APACHE II score categories

Group	APACHE II score	Patients, No.	14-Day Mortality (%)	Adjusted OR^a^ (95% CI)	*P*-value
I	<=15	43	16.3	2.42 (0.38–15.18)	0.345
II	16–25	108	26.9	0.83 (0.33–2.13)	0.704
III	26–35	110	51.8	0.61 (0.26–1.40)	0.241
IV	> = 36	75	88.0	0.0098 (0.0005–0.1885)	0.002

Abbreviations: *APACHE II* Acute Physiology and Chronic Health Evaluation II, *CI* confidence interval, *OR* odds ratio

^a^Adjusted for cerebrovascular accident, immunosuppressant use, recent surgery, extensive drug resistance, APACHE II score, and appropriate therapy

**Fig. 1:**
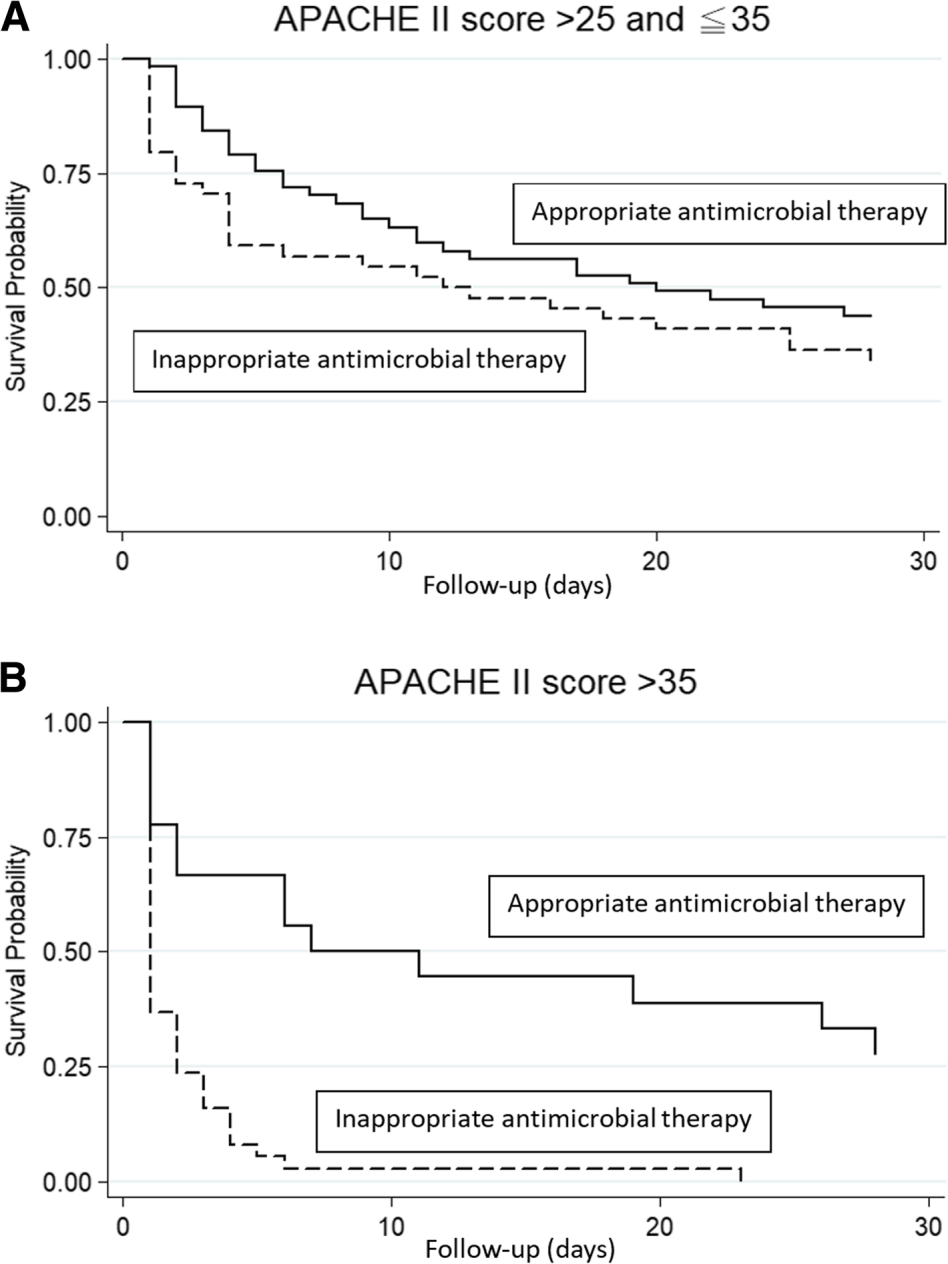
Kaplan-Meier survival curves at 28 days after *Acinetobacter baumannii* bacteremic pneumonia onset for patient receiving appropriate or inappropriate antimicrobial therapy, stratified by severity of infection. A, Group III, with Acute Physiology and Chronic Health Evaluation II scores >25 and ≤35. B, Group IV, with APACHE II score >35. Abbreviation: APACHE II, Acute Physiology and Chronic Health Evaluation II

Kaplan-Meier survival curves were used to compare the impacts of receiving appropriate or inappropriate antimicrobial therapy on mortality, stratified by APACHE II score groups as mentioned in Table 3. Although no significant differences in survival were noted between patients receiving appropriate versus inappropriate antimicrobial therapy in group I (*p* = 0.7106, by log-rank test), II (*p* = 0.9843, by log-rank test) (figures not shown), and III (*p* = 0.2014, by log-rank test) (Fig. 1a), there was a significant advantage in survival for appropriate compared to inappropriate use of antimicrobial therapy in group IV (*p* < 0.001, by log-rank test) (Fig. 1b).

The effect of appropriate antimicrobial therapy on 28-day survival was analyzed as per the above analyses. The results were similar to those found when using all-cause 14-day mortality as the primary outcome measure (data not shown).

The multivariate analysis of the demographic and clinical characteristics between patients receiving appropriate (151 patients) or inappropriate (185 patients) antimicrobial therapy showed that those with a history of myocardial infarction (*p* = 0.045), higher APACHE II scores (*p* = 0.001), and extensive drug resistance (*p* = 0.029) were more likely to receive inappropriate antimicrobial therapy. On the other hand, patients who has history of connective tissue disease (*p* = 0.035) and received a central venous catheter at the time of bacteremia onset (*p* = 0.004) had a higher chance of receiving appropriate antibiotics.

The independent impact of appropriate antimicrobial therapy on 14-day mortality were further explored by including age, gender, extensive drug resistance, APACHE II score and all the comorbidities in Table 1 in the multivariable logistic regression model (Supplemental Table S3). The results were similar to those including the statistically significant predictors using stepwise logistic regression (Table 2). Further analysis showed that the interaction between APACHE II score and appropriate antimicrobial therapy was statistically significant after adjusting for the demographic characteristics and comorbidities (Supplemental Table S4). The results were similar to the analysis using the statistically significant predictors in the regression model (Table 3), demonstrating that appropriate antimicrobial therapy has an independent association with a lower 14-day mortality of the most severely ill patients.

Both appropriate and inappropriate antimicrobials prescribed to patients are analyzed (Tables 4 and 5, respectively) and APACHE II scores among patient groups receiving different regimens were not significantly different. Among patients who received appropriate antimicrobial therapy, those receiving tigecycline-based or colistin-based therapy had a higher 14-day and 28-day mortality (Table 4), and no antimicrobial class was associated with a higher or lower 14-day and 28-day mortality after a multivariable analysis (data now shown). Patients receiving tigecycline-based or colistin-based therapy had been infected *carbapenem-resistant A. baumannii* (CRAB) more frequently than those receiving other antimicrobial agents (97.1% vs 57.3%, *p* < 0.001). Among patients who received inappropriate antimicrobial therapy, patients receiving antipseudomonal penicillins had a higher 28-day mortality compared to other antimicrobial therapies after multivariable adjustment (data not shown). For patients infected with CRAB receiving appropriate antimicrobial therapy, carbapenem + tigecycline-based therapy was associated with a higher 14-day and 28-day mortality (univariate analysis, Supplemental Table S5), but no antimicrobial class was associated with a higher or lower 14- or 28-day mortality after multivariable adjustment. For patients infected with CRAB receiving inappropriate antimicrobial therapy, no antimicrobial class was associated with a higher or lower 14- or 28-day mortality (Supplemental Table S6).

**Table 4:** Antimicrobial regimens for the treatment of *Acinetobacter baumannii* bacteremic pneumonia (appropriate antibiotics)

Main agents used^a,b^	No. (%) of patients (*n* = 151)	APACHE II score, median (IQR)^d^	No. (%) of patients				
			Combination therapy^e^	14-Day Mortality	*P*-value	28-Day Mortality	*P*-value
Anti-pseudomonas penicillin-based therapy	13 (8.6)	245 (19–29)	8 (61.5)	6 (46.2)	0.513	8 (61.5)	.319
Anti-pseudomonas cephalosporin-based therapy	31 (20.5)	25 (19–32)	24 (77.4)	10 (32.3)	0.479	12 (38.7)	.229
Carbapenem-based therapy	59 (39.1)	25 (18–28)	25 (42.4)	20 (33.9)	0.434	28 (47.5)	.861
Colistin-based therapy	55 (36.4)	25 (19–29)	49 (89.1)	28 (50.9)	0.012	33 (60.0)	.030
Tigecycline-based therapy	54 (35.8)	28 (22–32)	46 (85.2)	28 (51.9)	0.008	32 (59.3)	.045
Fluoroquinolone-based therapy	7 (4.6)	25 (12–36)	3 (42.9)	4 (57.1)	0.278	4 (57.1)	.633
Sulbactam-based therapy	24 (15.9)	26 (22–28)	13 (54.2)	7 (29.2)	0.344	10 (41.7)	.475
Carbapenem + colistin-based therapy	15 (9.9)	24 (18–28)	6 (40.0)	9 (60.0)	0.061	11 (73.3)	.041
Carbapenem + tigecycline-based therapy	12 (8.0)	25 (22–28)	7 (58.3)	9 (75.0)	0.006	10 (83.3)	.011
Carbapenem + sulbactam-based therapy	6 (4.0)	25 (22–28)	5 (83.3)	4 (66.7)	0.200	5 (83.3)	.107
Colistin + tigecycline-based therapy	25 (16.6)	27 (20–32)	12 (48.0)	14 (56.0)	0.039	15 (60.0)	.202
Carbapenem + colistin + tigecycline-based therapy	4 (2.7)	24 (20–28)	2 (50.0)	4 (100.0)	0.019	4 (100.0)	.052
Antimicrobial regimens^c^							
Anti-pseudomonas penicillin only	5 (3.3)	24 (19–30)		2 (40.0)	1.000	4 (80.0)	.198
Anti-pseudomonas cephalosporin only	7 (4.6)	23 (16–30)		2 (28.6)	0.711	2 (28.6)	.444
Carbapenem + colistin	9 (6.0)	24 (18–26)		4 (44.4)	0.730	6 (66.7)	.315
Carbapenem + tigecycline	5 (3.3)	25 (26–28)		3 (60.0)	0.366	3 (60.0)	.673
Carbapenem + sulbactam	1 (0.7)	17		0	1.000	0	1.000
Carbapenem + tigecycline + colistin	2 (1.3)	17, 22		2 (100)	0.141	2 (100)	.232
Tigecycline only	8 (5.3)	29 (28–33)		5 (62.5)	0.155	5 (62.5)	.484
Colistin + tigecycline	13 (8.6)	29 (22–38)		7 (53.9)	0.210	7 (53.9)	.678

^a^An antimicrobial agent (or antimicrobial agents)-based therapy denotes the corresponding antimicrobial agent(s) alone or in combination with other antimicrobial agent(s)

^b^“Colistin” denotes intravenous colistin only. Inhaled colistin is not included

^c^Not in combination with other antimicrobial agents

^d^IQR, interquartile range. When the case number is less than 4, the APACHE II score for each case is shown

^e^Combination therapy is defined as administration of more than one antimicrobial agent

**Table 5:** Antimicrobial regimens for the treatment of *Acinetobacter baumannii* bacteremic pneumonia (inappropriate antibiotics)

Main agents used^**a,b**^	No. (%) of patients (***n*** = 185)	APACHE II score, median (IQR)^**d**^	No. (%) of patients				
			Combination therapy^e^	14-Day Mortality	*P*-value	28-Day Mortality	*P*-value
Anti-pseudomonas penicillin-based therapy	28 (15.1)	33 (24–39)	3 (10.7)	19 (67.9)	.142	22 (78.6) ^f^	.077
Anti-pseudomonas cephalosporin-based therapy	36 (19.5)	31 (21–40)	8 (22.2)	17 (47.2)	.287	22 (61.1)	.710
Carbapenem-based therapy	61 (33.0)	29 (24–38)	11 (18.0)	38 (62.3)	.170	45 (73.8)	.047
Colistin-based therapy	7 (3.8)	28 (21–30)	7 (100)	3 (42.9)	.702	6 (85.7)	.425
Tigecycline-based therapy	13 (7.0)	33 (25–39)	10 (76.9)	9 (69.2)	.390	10 (76.9)	.382
Fluoroquinolone-based therapy	10 (5.4)	28 (20–39)	3 (30.0)	5 (50.0)	.737	7 (70.0)	1
Sulbactam-based therapy	10 (5.4)	30 (18–37)	6 (60.0)	5 (50.0)	.737	7 (70.0)	1
Carbapenem + colistin-based therapy	3 (1.6)	19, 27, 28	1 (33.3)	0	.088	3 (100)	.555
Carbapenem + tigecycline-based therapy	4 (2.2)	27 (22–31)	2 (50.0)	2 (50.0)	1	3 (75.0)	1
Carbapenem + sulbactam-based therapy	5 (2.7)	31 (29–31)	3 (60.0)	3 (60.0)	1	4 (80.0)	.655
Colistin + tigecycline-based therapy	2 (1.1)	19, 40	1 (50.0)	1 (50.0)	1	2 (100)	.535
Carbapenem + colistin + tigecycline-based therapy	1 (0.5)	19	0	0	.449	1 (100)	1
Antimicrobial regimens^c^							
Anti-pseudomonas penicillin only	25 (13.5)	33 (24–39)		18 (72.0)	.068	20 (80.0)	.070
Anti-pseudomonas cephalosporin only	28 (15.1)	32 (22–41)		14 (50.0)	.553	17 (60.0)	.714
Carbapenem + colistin	2 (1.1)	27, 28		0	.200	2 (100.0)	.535
Carbapenem + tigecycline	2 (1.1)	25, 33		1 (50.0)	1	1 (50.0)	1
Carbapenem + sulbactam	2 (1.1)	31, 40		2 (100)	.503	2 (100)	.535
Carbapenem + fluoroquinolone	1 (0.5)	43		1 (100)	1	1(100)	1
Carbapenem + tigecycline + colistin	1 (0.5)	19		0	.449	1(100)	1
Tigecycline only	3 (1.6)	30, 33, 38		3 (100)	.254	3 (100)	.555
Colistin + tigecycline	1 (0.5)	40		1 (100)	1	1 (100)	1

^a^An antimicrobial agent (or antimicrobial agents)-based therapy denotes the corresponding antimicrobial agent(s) alone or in combination with other antimicrobial agent(s)

^b^“Colistin” denotes intravenous colistin only. Inhaled colistin is not included

^c^Not in combination with other antimicrobial agents

^d^IQR, interquartile range. When the case number is less than 4, the APACHE II score for each case is shown

^e^Combination therapy is defined as administration of more than one antimicrobial agent

^f^Patients receiving antipseudomonal penicillin therapy had a significantly higher 28-day mortality compared to other antimicrobial therapy after multivariable adjustment

## Discussion

The efficacy of antimicrobial therapy for *A. baumannii* bacteremic pneumonia has been difficult to establish due to the three phenotypically indistinguishable *Acinetobacter* species that make up the Ab group and the confounding influence of underlying diseases and severity of infection. This retrospective study analyzed the effect of appropriate antimicrobial therapy on the 14-day mortality of the patients with genomically identified monomicrobial *A. baumannii* bacteremic pneumonia after adjusting for multiple risk factors. We demonstrated that appropriate antimicrobial therapy lowers mortality in the most severely ill patients.

The impacts of appropriate antimicrobial therapy on patients might be modified by the illness severity. It has been found that inappropriate antimicrobial therapy seem to do less harm in non-severe cases and in the most severely ill patients with short life expectancies [26]. On the other hand, our previous study of *A. baumannii* bacteremia patients showed that appropriate antimicrobial therapy reduced mortality in severely ill patients (APACHE II score > 25) [18].. Another study on carbapenem nonsusceptible *Klebsiella pneumoniae* also suggested that appropriate antimicrobial therapy did not benefit non-severe patients (APACHE II < 15) [27]. Our observation that appropriate antimicrobial therapy may be of crucial importance to the survival of the most severely ill patients (APACHE II score > 35) is in line with the previous studies. To our knowledge, this study is the first to explore the influence of severity of illness on the impacts of appropriate antimicrobial therapy in *A. baumannii* bacteremic pneumonia patients.

The pneumonia caused by phenotypically identified“*A. baumannii*” described in many studies actually comprises pneumonia caused by either one of the *Acinetobacter* species in the Ab group [13, 28]. There are differences in antimicrobial resistance and outcomes between *A. baumannii* and other *Acinetobacter* species in the Ab group [29–31], therefore pneumonia caused by these different *Acinetobacter* species cannot be considered as a single clinical entity. This study separated *A. baumannii* from other *Acinetobacter* species to avoid the confounding effect caused by the inclusion of a mixture of *Acinetobacter* species.

Colistin and tigecycline are often used for treatment of carbapenem-resistant *Acinetobacter* infections or as a salvage therapy for *Acinetobacter* infections with carbapenem treatment failure. However, our results showed that patients receiving tigecycline-based or colistin-based therapy, even both appropriate, still had a high 14- and 28-day mortality. Admittedly, patients receiving tigecycline-based or colistin-based therapy were more likely to have had been infected by CRAB strains and CRAB infection were associated with poorer outcome. However, tigecycline-based or colistin-based therapy was still not associated with a lower 14- and 28-day mortality for patients infected with CRAB. Colistin is administered as an inactive prodrug (colistin methanesulfonate) which results in a prolonged period of low plasma concentrations of the active drug and thereby influences its efficacy [32]. Possible explanations for tigecycline include its bacteriostatic property, a low AUC/MIC ratio [33–37], pneumonia as a source of bacteremia [36, 38], and relatively high MICs of tigecycline of our study isolates that were unachievable by the currently approved dose of tigecycline in the serum [21].

Among patients who received inappropriate antimicrobial therapy, patients receiving antipseudomonal penicillins had a higher 28-day mortality compared to other antimicrobial therapies. The similarity of APACHE II scores between patient groups receiving different regimens excludes disease severity as a confounder to explain the difference in mortality. Our finding suggested the potential detrimental effect of antipseudomonal penicillins for the treatment of *A. baumannii* bacteremic pneumonia. Further investigation is warranted to explore the cause of the finding.

Our study had some limitations. First, it is a retrospective study which is prone to selection bias and may limit the generalizability of our study. Second, our study included only patients with bacteremic pneumonia, thus the findings may not be applicable to *A. baumannii* pneumonia patients without bacteremia. The strengths of our study include the large case numbers obtained from multiple medical centers, genomically defined *A. baumannii,* and the adjustment of various risk factors.

## Conclusion

Appropriate antimicrobial therapy decreases the 14-day mortality of the most severely ill patients with *A. baumannii* bacteremic pneumonia. Further research is needed to determine the most effective antimicrobial therapy for *A. baumannii* bacteremic pneumonia.

## Supplementary information

**Additional file 1: Table S1.** Adjusted odds ratios for appropriate antibiotics for 14-day mortality in patients with *Acinetobacter baumannii* bacteremic pneumonia: Stratified by APACHE II Score in quartiles. **Table S2.** Logistic regression of predictors for 14-day mortality in low APACHE score patients (APACHE Score < 36) with *Acinetobacter baumannii* bacteremic pneumonia. **Table S3.** Logistic regression of demographic characteristics and comorbidities for 14-day mortality in patients with *Acinetobacter baumannii* bacteremic pneumonia. **Table S4.** Odds ratios adjusting for gender and comorbidities for appropriate antibiotics for 14-day mortality in patients with *Acinetobacter baumannii* bacteremic pneumonia: Stratified by APACHE II score categories. **Table S5.** Antimicrobial regimens for the treatment of carbapenem-resistant *Acinetobacter baumannii* bacteremic pneumonia (appropriate antibiotics). **Table S6.** Antimicrobial regimens for the treatment of carbapenem-resistant *Acinetobacter baumannii* bacteremic pneumonia (inappropriate antibiotics).  

## Data Availability

All data generated or analyzed during this study are included in this published article and its supplementary information files. Original data are available from the corresponding author upon reasonable request.

## Abbreviations

*A. baumannii*: *Acinetobacter baumannii*
APACHE: Acute Physiology and Chronic Health Evaluation
AUC: Area under the curve
BAL: Bronchoalveolar lavage
CI: Confidence interval
CRAB: Carbapenem-resistant *Acinetobacter baumannii*
CCH: Changhua Christian Hospital
ICU: Intensive care unit
MDR: Multiple drug resistant
MIC: Minimum inhibitory concentration
MMH: Mackay Memorial Hospital
NTUH: National Taiwan University Hospital
OR: Odds ratio
TSGH: Tri-Service General Hospital
TVGH: Taipei Veterans General Hospital
XDR: Extensively drug resistant
