# Supplementary material for: Influence of severity of infection on the effect of appropriate antimicrobial therapy for Acinetobacter baumannii bacteremic pneumonia
Source: Antimicrob Resist Infect Control. 2020 Sep 29;9:160. doi: 10.1186/s13756-020-00824-4 (PMC7523485; doi:10.1186/s13756-020-00824-4)
Supplement: Supplementary file 1 — Additional file 1: Table S1. Adjusted odds ratios for appropriate antibiotics for 14-day mortality in patients with Acinetobacter baumannii bacteremic pneumonia: Stratified by APACHE II Score in quartiles. Table S2. Logistic regression of predictors for 14-day mortality in low APACHE score patients (APACHE Score < 36) with Acinetobacter baumannii bacteremic pneumonia. Table S3. Logistic regression of demographic characteristics and comorbidities for 14-day mortality in patients with Acinetobacter baumannii bacteremic pneumonia. Table S4. Odds ratios adjusting for gender and comorbidities for appropriate antibiotics for 14-day mortality in patients with Acinetobacter baumannii bacteremic pneumonia: Stratified by APACHE II score categories. Table S5. Antimicrobial regimens for the treatment of carbapenem-resistant Acinetobacter baumannii bacteremic pneumonia (appropriate antibiotics). Table S6. Antimicrobial regimens for the treatment of carbapenem-resistant Acinetobacter baumannii bacteremic pneumonia (inappropriate antibiotics). [file 13756_2020_824_MOESM1_ESM.docx]

**Supplemental material**

**Table S1**. **Adjusted odds ratios for appropriate antibiotics for 14-day mortality in patients with *Acinetobacter baumannii* bacteremic pneumonia: Stratified by APACHE II Score in quartiles**

| Group | APACHE II  score | Patients,  No. | 14-Day  Mortality (%) | Adjusted OR (95% CI) | P-value |
| --- | --- | --- | --- | --- | --- |
| I | ≤20 | 86 | 12.0 | 2.30 (0.77-6.92) | .137 |
| II | 21-27 | 101 | 17.8 | 1.37 (0.50-3.72) | .54 |
| III | 28-34 | 65 | 25.2 | 0.42 (0.12-1.43) | .166 |
| IV | ≥35 | 84 | 45.3 | 0.025 (0.003-0.183) | <0.001 |

Abbreviations: APACHE II, Acute Physiology and Chronic Health Evaluation II; CI, confidence interval; OR, odds ratio.

**Table S2. Logistic regression of predictors for 14-day mortality in low APACHE score patients (APACHE Score <36) with *Acinetobacter*** ***baumannii* bacteremic pneumonia**

|  | Univariable Analysis | |  | Multivariable Analysis | |
| --- | --- | --- | --- | --- | --- |
| Characteristics | OR (95% CI) | P-value |  | OR (95% CI) | P-value |
| Hematologic malignancy | 3.09 (1.15-8.26) | 0.025 |  |  |  |
| Cerebrovascular accident | 0.43 (0.21-0.87) | 0.018 |  | 0.42 (0.19-0.92) | 0.029 |
| Immunosuppressant use | 2.70 (1.54-4.75) | 0.001 |  | 1.89 (1.00-3.58) | 0.05 |
| Recent surgery | 0.46 (0.25-0.82) | 0.009 |  | 0.46 (0.24-0.88) | 0.009 |
| Carbapenem resistance | 2.65 (1.47-4.78) | 0.001 |  |  |  |
| Extensive drug resistance | 3.23 (1.90-5.47) | <0.001 |  | 3.16 (1.78-5.63) | <0.001 |
| Previous ventilator use | 1.78 (1.06-2.96) | 0.028 |  |  |  |
| APACHE II score | 1.11 (1.06-1.16) | <0.001 |  | 1.08 (1.03-1.13) | 0.001 |
| Appropriate antimicrobial therapy | 0.549 (0.52-1.42) | 0.549 |  |  |  |

All biologically plausible variables with a p-value <0.05 in the univariable analysis were considered for inclusion in the logistic regression model in the multivariable analysis. A stepwise selection process was utilized. We found that only cerebrovascular accident, immunosuppressant use, recent surgery, extensive drug resistance, and APACHE II score were statistically significant factors for 14-day mortality.

Abbreviations: APACHE II, Acute Physiology and Chronic Health Evaluation II; CI, confidence interval

**Table S3. Logistic regression of demographic characteristics and comorbidities for 14-day mortality in patients with *Acinetobacter baumannii* bacteremic pneumonia**

| Characteristic | Multivariable Analysis | |
| --- | --- | --- |
|  | OR (95% CI) | P value |
| Male | 1.72 (0.89-3.34) | 0.108 |
| Age | 1.00 (0.98-1.02) | 0.875 |
| Cerebrovascular accident | 0.45 (0.22-0.94) | 0.034 |
| Hypertension | 0.75 (0.41-1.36) | 0.337 |
| Type 2 diabetes mellitus | 0.79 (0.43-1.47) | 0.459 |
| Congestive heart failure | 0.84 (0.40-1.73) | 0.631 |
| Myocardial infarction | 0.84 (0.38-1.84) | 0.656 |
| Chronic obstructive pulmonary disease | 0.78 (0.39-1.60) | 0.504 |
| Chronic kidney disease | 0.87 (0.48-1.59) | 0.656 |
| Liver cirrhosis | 0.77 (0.30-2.00) | 0.589 |
| Collagen vascular disease | 1.88 (0.64-5.56) | 0.253 |
| Hematologic malignancy | 1.05 (0.24-4.55) | 0.947 |
| Solid tumor | 1.18 (0.28-4.90) | 0.822 |
| Immunosuppressant use | 1.53 (0.76-3.07 | 0.23 |
| Chemotherapy | 0.84 (0.25-2.82) | 0.782 |
| Neutropenia | 1.02 (0.29-3.63) | 0.972 |
| Recent surgery | 0.45 (0.24-0.86) | 0.015 |
| Extensive drug resistance | 3.63 (2.02-6.52) | <0.001 |
| APACHE II score (categorical) | 3.39 (2.42-4.75) | <0.001 |
| Appropriate antimicrobial therapy | 0.53 (0.31-0.93) | 0.027 |

All biologically plausible variables were included in the logistic regression model in the multivariable analysis. We found that only cerebrovascular accident, recent surgery, extensive drug resistance, APACHE II score, and appropriate therapy were statistically significant factors for 14-day mortality.

Abbreviations: APACHE II, Acute Physiology and Chronic Health Evaluation II; CI, confidence interval

**Table S4. Odds ratios adjusting for gender and comorbidities for appropriate antibiotics for 14-day mortality in patients with *Acinetobacter baumannii* bacteremic pneumonia: Stratified by APACHE II score categories**

| Group | APACHE II  score | Patients,  No. | 14-Day  Mortality (%) | Adjusted OR^a^ (95% CI) | P-value |
| --- | --- | --- | --- | --- | --- |
| I | <=15 | 43 | 16.3 | 2.77 (0.42-18.42) | 0.291 |
| II | 16-25 | 108 | 26.9 | 1.10 (0.43-2.80) | 0.841 |
| III | 26-35 | 110 | 51.8 | 0.55 (0.24-1.27) | 0.159 |
| IV | >=36 | 75 | 88.0 | 0.0189 (0.0017-0.2091) | 0.001 |

^a^Adjusted for male, recent surgery, extensive drug resistance, Charlson comorbidity index, APACHE II score, and appropriate therapy. Age is included in the Charlson comorbidity index.

Abbreviations: APACHE II, Acute Physiology and Chronic Health Evaluation II; CI, confidence interval; OR, odds ratio.

**Table S5. Antimicrobial regimens for the treatment of** **carbapenem-resistant *Acinetobacter baumannii* bacteremic pneumonia (appropriate antibiotics)**

| Main agents used*^a,b^* | No. (%) of patients  (*n* = 100) | APACHE II score, median (IQR)*^d^* | No. (%) of patients | | | | |
| --- | --- | --- | --- | --- | --- | --- | --- |
|  |  |  | Combination therapy^e^ | 14-Day Mortality | P-value | 28-Day Mortality | P-value |
| Anti-pseudomonas penicillin-based therapy | 9 (9.0) | 21 (19-29) | 7 (77.8) | 4 (44.4) | .922 | 5 (55.6) | .972 |
| Anti-pseudomonas cephalosporin-based therapy | 21 (21.0) | 25 (20-32) | 20 (95.2) | 7 (33.3) | .190 | 9 (42.9) | .208 |
| Carbapenem-based therapy | 28 (28) | 26 (19-28) | 24 (85.7) | 14 (50.0) | .617 | 17 (60.7) | .474 |
| Colistin-based therapy | 55 (55.0) | 26 (19-29) | 49 (89.1) | 28 (50.9) | .276 | 33 (60.0) | .267 |
| Tigecycline-based therapy | 53 (53.0) | 28 (22-32) | 45 (84.9) | 28 (52.8) | .146 | 32 (60.4) | .251 |
| Fluoroquinolone-based therapy | 4 (4.0) | 33 (19-40) | 2 (50.0) | 3 (75.0) | .235 | 3 (75.0) | .412 |
| Sulbactam-based therapy | 16 (16.0) | 27 (22-30) | 9 (56.3) | 6 (37.5) | .457 | 8 (50.0) | .661 |
| Carbapenem + colistin-based therapy | 15 (15.0) | 25 (18-28) | 6 (40.0) | 9 (60.0) | .238 | 11 (73.3) | .122 |
| Carbapenem + tigecycline-based therapy | 12 (12.0) | 27 (22-28) | 7 (58.3) | 9 (75.0) | .032 | 10 (83.3) | .035 |
| Carbapenem + sulbactam-based therapy | 5 (5.0) | 27 (27-28) | 5 (100.0) | 4 (80.0) | .177 | 5 (100.0) | .062 |
| Colistin + tigecycline-based therapy | 25 (25.0) | 27 (20-32) | 12 (48.0) | 14 (56.0) | .247 | 15 (60.0) | .562 |
| Carbapenem + colistin + tigecycline-based therapy | 4 (4.0) | 25 (20-28) | 2 (50.0) | 4 (100.0) | .042 | 4 (100.0) | .125 |
| Antimicrobial regimens*^c^* |  |  |  |  |  |  |  |
| Anti-pseudomonas penicillin only | 2 (2.0) | 25 (19-30) |  | 1 (50.0) | 1.000 | 2 (100.0) | .500 |
| Anti-pseudomonas cephalosporin only | 1 (1.0) | 33 |  | 0 (0) | 1.000 | 0 (0) | .450 |
| Carbapenem + colistin | 9 (9.0) | 25 (18-26) |  | 4 (44.4) | 1.000 | 6 (66.7) | .508 |
| Carbapenem + tigecycline | 5 (5.0) | 27 (26-28) |  | 3 (60.0) | .659 | 3 (60.0) | 1.000 |
| Carbapenem + tigecycline + colistin | 2 (2.0) | 20 (17-22) |  | 2 (100.0) | .209 | 2 (100.0) | .500 |
| Tigecycline only | 8 (8.0) | 32 (28-33) |  | 5 (62.5) | .465 | 5 (62.5) | .727 |
| Colistin + tigecycline | 13 (13.0) | 29 (22-28) |  | 7 (53.9) | .543 | 7 (53.9) | .929 |

*^a^*An antimicrobial agent (or antimicrobial agents)-based therapy denotes the corresponding antimicrobial agent(s) alone or in combination with other antimicrobial agent(s).

*^b^*“Colistin” denotes intravenous colistin only. Inhaled colistin is not included.

*^c^*Not in combination with other antimicrobial agents.

*^d^*IQR, interquartile range. When the case number is less than 4, the APACHE II score for each case is shown.

*^e^*Combination therapy is defined as administration of more than one antimicrobial agent.

**Table S6. Antimicrobial regimens for the treatment of carbapenem-resistant *Acinetobacter baumannii* bacteremic pneumonia (inappropriate antibiotics)**

| Main agents used*^a,b^* | No. (%) of patients  (*n* = 133) | APACHE II score, median (IQR)*^e^* | No. (%) of patients | | | | |
| --- | --- | --- | --- | --- | --- | --- | --- |
|  |  |  | Combination  therapy*^e^* | 14-Day  Mortality | P-value | 28-Day  Mortality | P-value |
| Anti-pseudomonas penicillin-based therapy | 22 (16.5) | 33 (24-40) | 3 (13.6) | 16 (72.7) | .274 | 19 (86.4) | .090 |
| Anti-pseudomonas cephalosporin-based therapy | 24 (18.1) | 31 (22-39) | 7 (29.2) | 11 (45.8) | .064 | 14 (58.3) | .117 |
| Carbapenem-based therapy | 55 (41.4) | 29 (24-38) | 10 (18.2) | 35 (63.6) | .806 | 42 (76.4) | .290 |
| Colistin-based therapy | 6 (4.5) | 29 (27-30) | 6 (100.0) | 3 (50.0) | .672 | 6 (100.0) | .182 |
| Tigecycline-based therapy | 12 (9.0) | 32 (24-40) | 10 (83.3) | 8 (66.7) | 1.000 | 9 (75.0) | 1.000 |
| Fluoroquinolone-based therapy | 8 (6.0) | 32 (24-41) | 3 (37.5) | 4 (50.0) | .474 | 5 (62.5) | .688 |
| Sulbactam-based therapy | 9 (6.8) | 31 (25-37) | 5 (55.6) | 5 (55.6) | .728 | 7 (77.8) | 1.000 |
| Carbapenem + colistin-based therapy | 3 (2.3) | 27 (19-28) | 1 (33.3) | 0 (0) | .051 | 3 (100.0) | .557 |
| Carbapenem + tigecycline-based therapy | 4 (3.0) | 27 (22-31) | 2 (50.0) | 2 (50.0) | .631 | 3 (75.0) | 1.000 |
| Carbapenem + sulbactam-based therapy | 4 (3.0) | 31 (30-36) | 2 (50.0) | 3 (75.0) | 1.000 | 4 (100.0) | .578 |
| Colistin + tigecycline-based therapy | 2 (1.5) | 30 (19-40) | 1 (50.0) | 1 (50.0) | 1.000 | 2 (100.0) | 1.000 |
| Carbapenem + colistin + tigecycline-based therapy | 1 (0.8) | 19 | 0 (0) | 0 (0) | .376 | 1 (100.0) | 1.000 |
| Antimicrobial regimens*^c^* |  |  |  |  |  |  |  |
| Anti-pseudomonas penicillin only | 19 (14.3) | 33 (24-43) |  | 15 (79.0) | .108 | 17 (89.5) | .060 |
| Anti-pseudomonas cephalosporin only | 17 (12.8) | 35 (24-39) |  | 8 (47.1) | .162 | 9 (52.9) | .071 |
| Carbapenem + colistin | 2 (1.5) | 28 (27-28) |  | 0 (0) | .140 | 2 (100.0) | 1.000 |
| Carbapenem + tigecycline | 2 (1.5) | 29 (25-33) |  | 1 (50.0) | 1.000 | 1 (50.0) | .491 |
| Carbapenem + sulbactam | 2 (1.5) | 36 (31-40) |  | 2 (100.0) | .527 | 2 (100.0) | 1.000 |
| Carbapenem + quinolone | 1 (0.8) | 43 |  | 1 (100.0) | 1.000 | 1 (100.0) | 1.000 |
| Carbapenem + tigecycline + colistin | 1 (0.8) | 19 |  | 0 (0) | .376 | 1 (100.0) | 1.000 |
| Tigecycline only | 2 (1.5) | 34 (30-38) |  | 2 (100.0) | .527 | 2 (100.0) | 1.000 |
| Colistin + tigecycline | 1 (0.8) | 40 |  | 1 (100.0) | 1.000 | 1 (100.0) | 1.000 |

*^a^*An antimicrobial agent (or antimicrobial agents)-based therapy denotes the corresponding antimicrobial agent(s) alone or in combination with other antimicrobial agent(s).

*^b^*“Colistin” denotes intravenous colistin only. Inhaled colistin is not included.

*^c^*Not in combination with other antimicrobial agents.

*^d^*IQR, interquartile range. When the case number is less than 4, the APACHE II score for each case is shown.

*^e^*Combination therapy is defined as administration of more than one antimicrobial agent
